# Diagnostic Value of Osteopontin in Ovarian Cancer: A Meta-Analysis and Systematic Review

**DOI:** 10.1371/journal.pone.0126444

**Published:** 2015-05-07

**Authors:** Zhi-De Hu, Ting-Ting Wei, Min Yang, Ning Ma, Qing-Qin Tang, Bao-Dong Qin, Hai-Tao Fu, Ren-Qian Zhong

**Affiliations:** 1 Department of Laboratory Diagnostics, Changzheng Hospital, The Second Military Medical University, Shanghai, China; 2 Department of Laboratory Medicine, The General Hospital, Ji’nan Military Region of PLA, Ji’nan, Shandong, China; Second University of Naples, ITALY

## Abstract

**Aims:**

Osteopontin (OPN) plays an important role in many physiological and pathological processes (wound healing, inflammation, immune response, and tumorigenesis). This meta-analysis assessed the diagnostic value of osteopontin in ovarian cancer.

**Methods and Results:**

Searches in Embase and PubMed were conducted, in order to identify eligible studies on osteopontin expression and its diagnostic value in ovarian cancer. The revised Quality Assessment for Studies of Diagnostic Accuracy (QUADAS-2) tool was applied to examine the quality of these studies and the overall osteopontin diagnostic accuracy in ovarian cancer was pooled using the bivariate model. The publication bias was assessed using funnel plots and Deek’s test. This search methodology resulted in 13 studies with a total of 839 ovarian cancer patients and 1439 controls in this meta-analysis. The overall osteopontin diagnostic sensitivity and specificity of ovarian cancer were 0.66 (95% CI, 0.51–0.78) and 0.88 (95% CI, 0.78–0.93), respectively. The area under summary receiver operating characteristic (sROC) curves (AUC) was 0.85 (95%CI, 0.81–0.88). There was no significant publication bias observed across the eligible studies. However, a major design deficiency of the eligible studies is the issue of subject selection bias.

**Conclusions:**

Osteopontin could be a useful biomarker in diagnosis of ovarian cancer. Due to the design deficits of the eligible studies, a future study with a larger sample size and better design is needed to rigorously confirm the diagnostic potential of osteopontin in ovarian cancer.

## Introduction

Ovarian cancer is a significant worldwide health problem in women [1–3], and globally accounted for more than 220,000 incident cases and approximately 160,000 cancer-related deaths in 2010 [4, 5]. Ovarian cancer is usually diagnosed at the advanced stages of disease and contributes to high mortality and a poor 5-year survival rate. Nevertheless, the 5-year survival rate in early ovarian cancer patients can reach up to 70–90% versus 17–36% in advanced ovarian cancer patients [6]. Therefore, timely and accurate diagnosis is critically important in improving survival of ovarian cancer patients. To date, histopathology examination is considered the gold standard in diagnosis of ovarian cancer, but the invasive nature in obtaining ovarian tissue has limited its application in early diagnosis of ovarian cancer because most ovarian cancer patients often have no visible symptoms. Thus, identfcation and evaluation of serum biomarkers could help the early diagnosis of this now lethal cancer. In this regard, transvaginal ultrasonography and serum levels of cancer antigen 125 (CA125) are the main tools to detect ovarian cancer early [7]. The former method requires a specific device and its diagnostic accuracy is largely affected by examiner experience. CA125, a member of the mucin glycoprotein family [8], is frequently used to detect ovarian cancer and approximately 90% of women with advanced ovarian cancer have elevated serum CA125 levels, while its diagnostic sensitivity and specificity are approximately 0.80 and 0.75, respectively [9]. Therefore, further studies of biomarkers for detection of ovarian cancer is promptly needed.

Osteopontin (OPN) is a secreted extracellular matrix glycoprotein and involved in a number of cellular processes, including wound healing, inflammation, the immune response, and tumorigenesis [10, 11]. In ovarian cancer, OPN is usually overexpressed, although it may also be overexpressed in other types of human cancer. Specifically, increase in serum OPN levels is often used to assess diagnosis and prognosis of various human cancers, such as malignant pleural mesothelioma [12], hepatocellular carcinoma [13] and breast cancer [14]. In diagnosis of ovarian cancer, OPN has been intensively studied; for example, two recent published meta-analyses [15, 16] showed that serum OPN levels were generally elevated in ovarian neoplasm patients, indicating that OPN is a potential diagnostic marker for ovarian cancer. However, the diagnostic characteristics such as sensitivity and specificity remain to be defined. Therefore, we performed a meta-analysis to ascertain: i) whether OPN is a useful tumor biomarker in ovarian cancer when used alone; ii) whether OPN can improve the accuracy of CA125 in diagnosis of ovarian cancer; and iii) whether and how future studies are needed to verify the diagnostic value of osteopontin for ovarian cancer.

## Materials and Methods

### Literature search

This meta-analysis was conducted and reported in accordance with the PRISMA guidelines for systematic reviews and meta-analyses (S1 PRISMA Checklist) [17]. Two investigators (ZD Hu and TT Wei) independently searched different databases, including PubMed and Embase to identify eligible studies that were published up to September 24, 2014. The search terms used for Pubmed were: “(osteopontin or OPN or SPP1 or secreted phosphoprotein) and ovarian”. A similar search strategy was used for Embase. Manual searches were also conducted by reviewing the references of the eligible studies on this topic. The titles and abstracts of the retrieved studies were independently reviewed to identify potentially eligible studies. If necessary, full-text of each publication was reviewed and any disagreement in selection of a study was resolved by full-text review.

### Inclusion and exclusion criteria

The inclusion criteria of the current systematic review and meta-analysis were: i) studies that evaluated the diagnostic accuracy of serum or plasma OPN in diagnosis of ovarian cancer; ii) sample size of ovarian cancer and non-ovarian cancer patients was more than 10, knowing that very small sample size studies may be vulnerable to selection bias; and iii) 2 x 2 tables could be constructed from the sensitivity and specificity reported or could be obtained from the receiver operating characteristic (ROC) curve. The exclusion criteria were applied to i) animal studies; ii) non-English publications; and iii) conference abstracts or letters to editors because they usually present limited data for analysis. For duplicate reports, only the study with more detailed information was included.

### Data extraction and quality assessment

The sample size, publication year, country origin of subjects, ovarian cancer and non-ovarian cancer patients, OPN test methods, reference, area under ROC curve (AUC), and threshold were extracted independently by two investigators (ZD Hu and TT Wei). A third investigator could intervene to resolve any discrepancies when the aforementioned reviewers disagreed. The true positive (TP), false-positive (FP), false-negative (FN), and true negative (TN) rates were calculated according to the sample size of ovarian cancer and non-ovarian cancer patients and the sensitivity and specificity reported using the following formulas: TP = number of ovarian cancer patients × sensitivity; FN = number of ovarian cancer patients × (1−sensitivity); TN = number of non-ovarian cancer patients × specificity; FP = number of non-ovarian cancer patients × (1−specificity). For studies that did not report sensitivity and specificity but displayed the ROC curve, we chose the point nearest to the upper left corner on the ROC curve as the optimal threshold, and the corresponding sensitivity and specificity were used for data extraction.

The quality of eligible studies was independently assessed using the revised Quality Assessment for Studies of Diagnostic Accuracy tool (QUADAS-2) [18]. The items or domains in QUADAS-2 were labeled as unknown if the corresponding design characteristics were not reported. Any disagreement in quality assessment was resolved by consensus.

### Statistical analyses

The overall OPN sensitivity and specificity in diagnosis of ovarian cancer were pooled using the bivariate model [19]. The bivariate model uses pairs of sensitivity and specificity as the starting point of the analysis and thus may be more reliable for estimating the diagnostic accuracy of index test in the meta-analysis, compared with the traditional summary receiver operating characteristic (sROC) approach that uses the diagnostic odds ratio (DOR) as the main outcome measure. The latter approach ignores the trade-off between sensitivity and specificity [19]. In addition, since the bivariate model uses a random effect approach for both specificity and sensitivity, the heterogeneity beyond chance could be regarded as a result of clinical and methodological differences among studies. The pooled positive and negative likelihood ratio was calculated according to the summary estimates of sensitivity and specificity. The funnel plots and the Deeks’s test were applied to assess the potential publication bias [20]. Univariate regression analysis was performed to explore the possible sources of heterogeneity across eligible studies. All analyses were performed using STATA 12.0 (Stata Corp LP, College Station, TX) and the midas command was used for all statistical analyses [21].

## Results

### Identification of eligible studies

**Fig 1:**
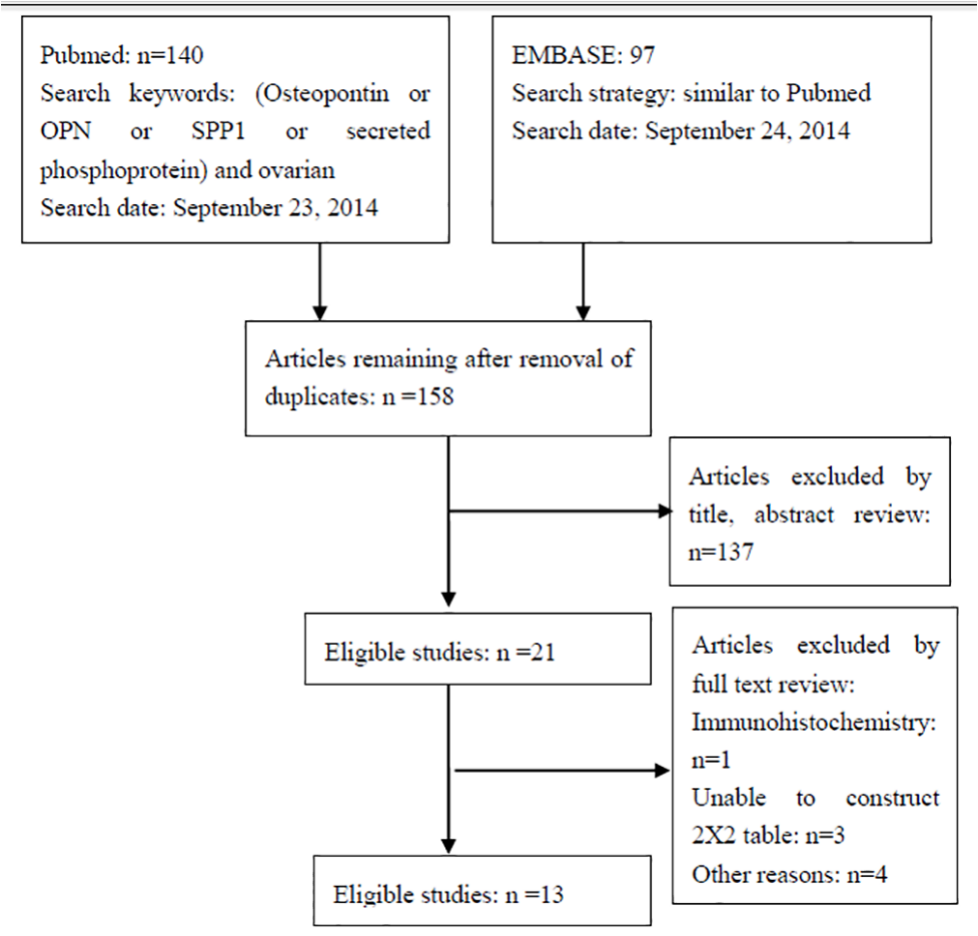
Flowchart of the systematic literature search and study selection process.

A flowchart depicting the study selection is shown in Fig 1. In this study, we found 13 studies eligible for meta-analysis [22–34] and the data are shown in Table 1. Among them, the sample size was arranged between 39 and 518, with a total sample size of 2278 (839 ovarian cancer patients and 1439 controls). Five studies explored the diagnostic accuracy of plasma OPN for ovarian cancer [22, 24, 28, 32, 34], while the remaining eight studies explored the diagnostic accuracy of serum OPN for ovarian cancer [23, 25–27, 29–31, 33]. The OPN test technique used was an ELISA, but the test kits were from different sources, such as IBL [22, 24, 26, 32] or R&D [25, 33], Milliplex MAP [30, 31], Multiplex PLA [28], and Beadlyte [27, 29], while one study did not detail the source [34]. For data collection, only one study reported that they were prospective [26] and another study reported that they were retrospective [27]. Most studies did not report how they collected data.

**Table 1:** Summary of these 13 eligible studies.

Author	Year	Country	No	OC/non-OC	FIGO grades	Reference	Matrix	Test method	Data collection
Kim [22]	2002	USA	251	51/200	NR	NR	Plasma	IBL	Unknown
Mor [23]	2005	USA	206	100/106	NR	NR	Serum	Assay Designs	Unknown
Nakae [24]	2006	Japan	127	32/95	NR	Pathology	Plasma	IBL	Unknown
Meinhold-Heerlein [25]	2007	Germany	149	67/67	7/2/49/9	NR	Serum	R&D	Unknown
Moore [26]	2008	USA	233	67/166	13/2/46/6	Pathology	Serum	IBL	Prospective
Visintin [27]	2008	USA	518	156/362	13/23/74/46	Clinical, surgical, histologic and pathologic	Serum	Beadlyte	Retrospective
Fredriksson [28]	2008	USA	39	19/20	4/0/10/5	NR	Plasma	Multiplex PLA	Unknown
Vrzalova [29]	2009	Czech	39	19/20	NR	NR	Serum	Beadlyte	Unknown
Lu [30]	2011	USA	304	151/153	11/6/98/35	NR	Serum	Milliplex MAP	Unknown
He [31]	2012	Canada	52	37/15	NR	Pathology	Serum	Milliplex MAP	Unknown
Bandiera [32]	2013	Italy	180	60/120	NR	NR	Plasma	IBL	Unknown
Moszynski [33]	2013	Poland	114	32/82	NR	Histopathological examination	Serum	R&D	Unknown
Milivojevic [34]	2013	Serbia	79	48/31	12/6/23/7	Histopathological examination	Plasma	NR	Unknown

NR: not reported. IBL: Immuno-Biological Laboratories. OC: ovarian cancer. FIGO, International Federation of Gynecology and Obstetrics.

### Diagnostic value of OPN in ovarian cancer assessed in individual studies

Table 2 showed the diagnostic performance of OPN in these eligible studies. Specifically, the AUC of the OPN levels were noted between 0.65 and 0.92 and the optimal threshold was noted between 2.91 ng/ml and 650 ng/ml. Moreover, the sensitivity was noted between 0.17 and 0.88, while the specificity arranged was recorded 0.54 and 1.00. Only one study clarified statistically that OPN and CA125 had comparable AUC under ROC curve, indicating that they had comparable diagnostic accuracy [33]. In addition, by using the net reclassification improvement (NRI) analysis [35], the data demonstrated that OPN could significantly improve the diagnostic accuracy of CA125 for ovarian cancer [33].

**Table 2:** Diagnostic value of OPN in individual studies of these 13 eligible publications.

Studies	AUCs (95%CI)	Thresholds	Sensitivity	Specificity	TP	FP	FN	TN
Kim [22]	NR	252 ng/ml	Early stage: 0.80	0.80	43	40	8	162
			Late stage: 0.85					
Mor [23]	NR	NR	0.80	0.76	80	25	20	81
Nakae [24]	NR	489 ng/ml	0.81	0.55	26	43	6	52
Meinhold-Heerlein [25]	NR	NR	0.88	1.00	59	0	8	67
Moore [26]	0.65(0.57–0.72)	NR	0.20	0.90	13	17	54	149
Visintin [27]	0.79(0.72–0.87)	NR	0.73	0.70	114	107	42	255
Fredriksson [28]	NR	NR	0.58	0.85	11	3	8	17
Vrzalova [29]	0.804	2.91 ng/ml	0.47	0.95	9	1	10	19
Lu [30]	NR	NR	0.17	0.99	26	1	125	152
He [31]	0.68	NR	0.65	0.66	24	5	13	10
Bandiera [32]	0.92(0.87–0.97)	NR	0.81	0.81	49	23	11	97
Moszynski [33]	0.83(0.75–0.90)	28.0 ng/ml	0.72	0.89	23	9	9	73
Milivojevic [34]	0.84(0.75–0.93)	650 ng/ml	0.63	0.90	30	3	18	28

NR, not reported;—, no data available; TP, true positive rate; FP, false-positive rate; FN, false-negative rate; TN, true negative rate.

### Quality assessment of these eligible studies

Table 3 lists the quality assessment of these 13 eligible studies. Specifically, the patient selection augmented the risk of bias and applicability concerns [22–25, 27, 28, 30] in seven studies due to the case-control study design. The index test domain in six studies [23, 27, 28, 30, 31, 33] was labeled as high because the diagnostic threshold was not pre-specified. The index domain in one study was labeled as unknown since there was no report of how the threshold had been chosen [25]. The follow-up and timing domain in four studies was labeled as high because of partial verification [24, 27, 28, 30].

**Table 3:** Quality assessment of these 13 eligible studies using QUADAS-2.

Study	Risk of bias				Applicability concerns		
	Patient selection	Index test	Reference standard	Flow and timing	Patient selection	Index test	Reference standard
Kim [22]	High	Low	Unknown	Unknown	High	Low	Unknown
Mor [23]	High	High	Unknown	Unknown	High	Low	Unknown
Nakae [24]	High	Low	Low	High	High	Low	Unknown
Meinhold-Heerlein [25]	High	Unknown	Unknown	Unknown	High	Low	Unknown
Moore [26]	Low	Low	Low	Low	Low	Low	Low
Visintin [27]	High	High	Low	High	High	Low	Low
Fredriksson [28]	High	High	Unknown	High	High	Low	Unknown
Vrzalova [29]	Unknown	Low	Unknown	Unknown	Unknown	Low	Unknown
Lu [30]	High	High	Unknown	High	High	Low	Unknown
He [31]	Unknown	High	Low	Unknown	Low	Low	Low
Bandiera [32]	Unknown	Low	Unknown	Unknown	Low	Low	Unknown
Moszynski [33]	Low	High	Low	Low	Low	Low	Low
Milivojevic [34]	Unknown	Low	Low	Unknown	Low	Low	Low

### Overall diagnostic value of OPN for ovarian cancer

**Fig 2:**
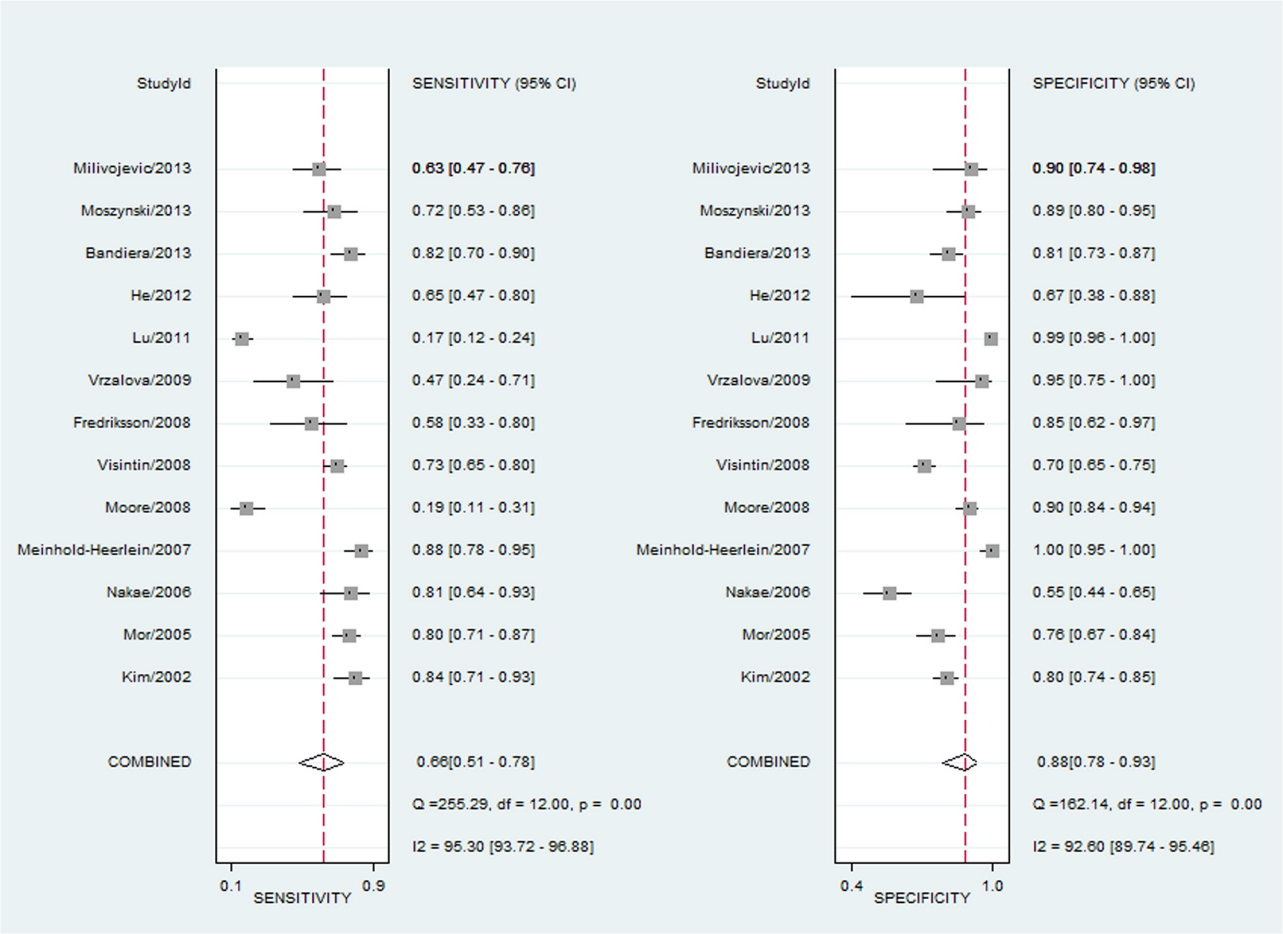
Sensitivity and specificity of OPN in diagnosis of ovarian cancer assessed by Forest plots.

**Fig 3:**
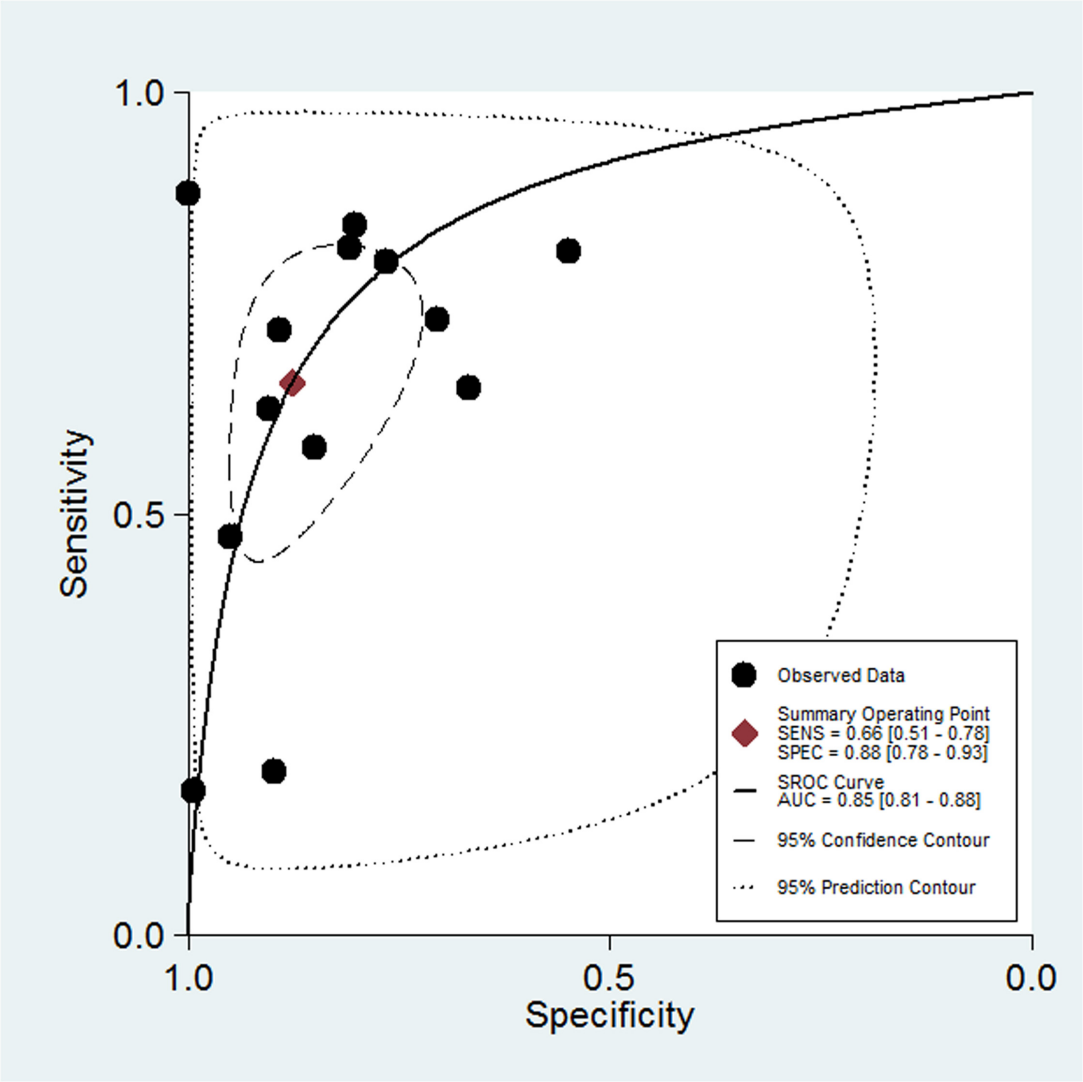
The summary receiver operating characteristic (sROC) curves (AUC) of OPN in diagnosis of ovarian cancer. The overall diagnostic efficiency is summarized by the regression curve.

Fig 2 shows the forest plot of diagnostic sensitivity and specificity of OPN for ovarian cancer patients. The overall diagnostic sensitivity and specificity of OPN were 0.66 (95% CI: 0.51–0.78) and 0.88 (95% CI: 0.78–0.93), respectively. In addition, the pooled PLR, NLR, diagnostic score and DOR were 5.30 (95% CI, 3.05–9.19), 0.39 (95%CI, 0.27–0.57), 2.60 (95%CI, 1.91–3.30) and 13.49 (95% CI, 6.75–26.97), respectively. Moreover, there was significant heterogeneity between the included studies, and the I^2^ for sensitivity and specificity was 95.30 (95%CI: 93.72–96.88) and 92.60 (95%CI: 89.74–95.46), respectively (Fig 2). Threshold effect analysis showed that there was only 26% of heterogeneity, which was likely to be due to a trade-off between sensitivity and specificity. AUC for OPN and ovarian cancer was 0.85 (95%CI, 0.81–0.88; Fig 3). Taken together, these results indicate that OPN is a useful biomarker in diagnosis of ovarian cancer.

### Univariate regression and subgroup analysis

**Fig 4:**
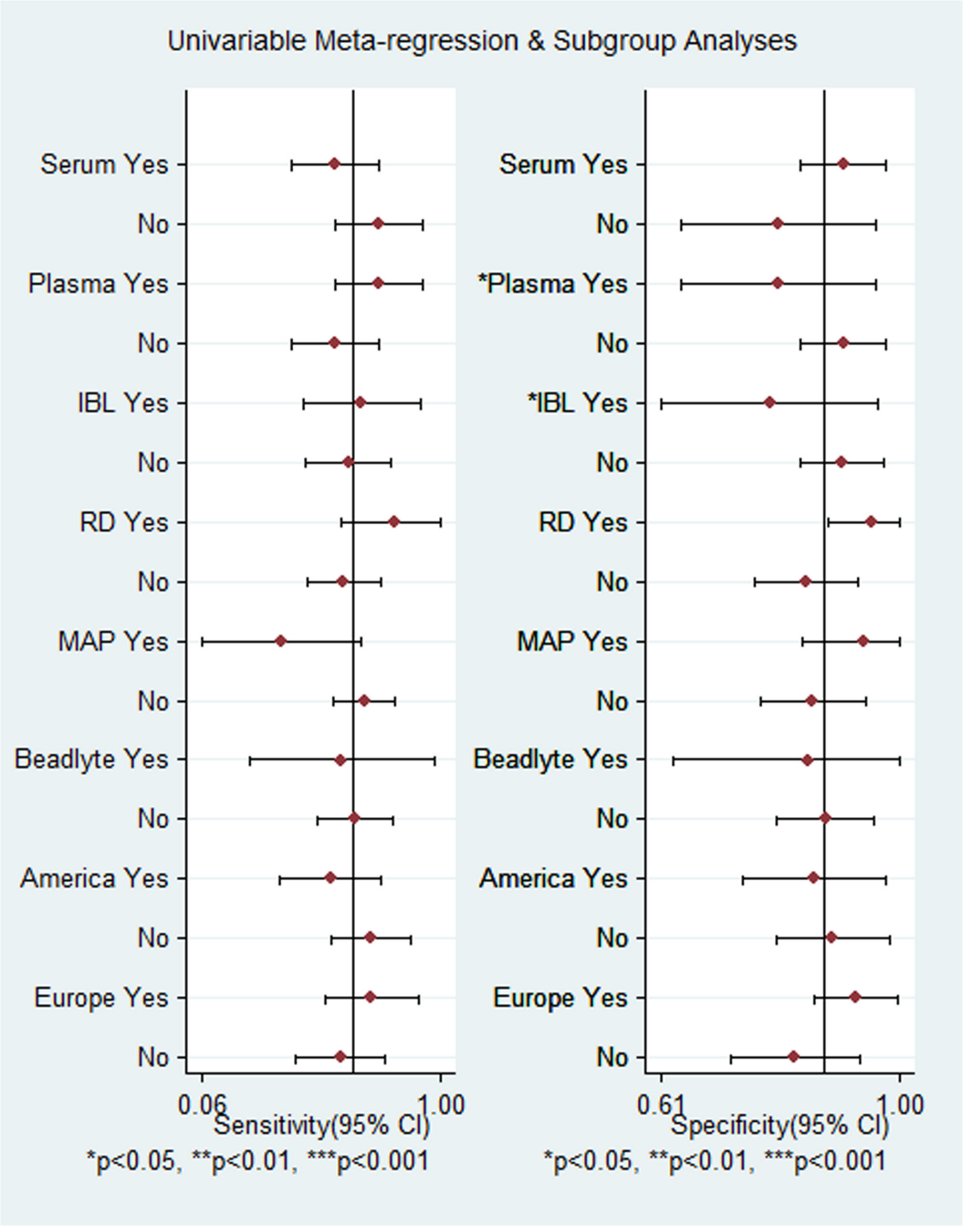
Subgroup analysis of OPN sensitivity and specificity in diagnosis of ovarian cancer.

Given the significant heterogeneity across these 13 eligible studies and only 26% of heterogeneity likely due to threshold effect, we performed a subgroup analysis and univariate meta-regression to explore the source of heterogeneity. Our hypothesis was that the test matrices (plasma or serum), test methods (IBL, R & D, MAP and Beadlyte) and country origin of the subjects (European or American) were the possible sources of heterogeneity; thus, a subgroup analysis was performed accordingly. As shown in Fig 4, plasma as the detection matrix and the IBL assay were used to determine OPN as the source of heterogeneity for specificity (*p* < 0.05 for both).

The threshold effect was not considered in the subgroup analysis, meaning that the effect of each covariate on specificity was estimated separately from that on sensitivity, and vice versa. Therefore, we performed a univariate meta-regression analysis to explore the possible sources of heterogeneity. The results showed that only Europe (I^2^ = 67%, *p* = 0.05) and R&D OPN kit (I^2^ = 75%, *p* = 0.02) were the sources of heterogeneity across these eligible studies.

### Publication bias

**Fig 5:**
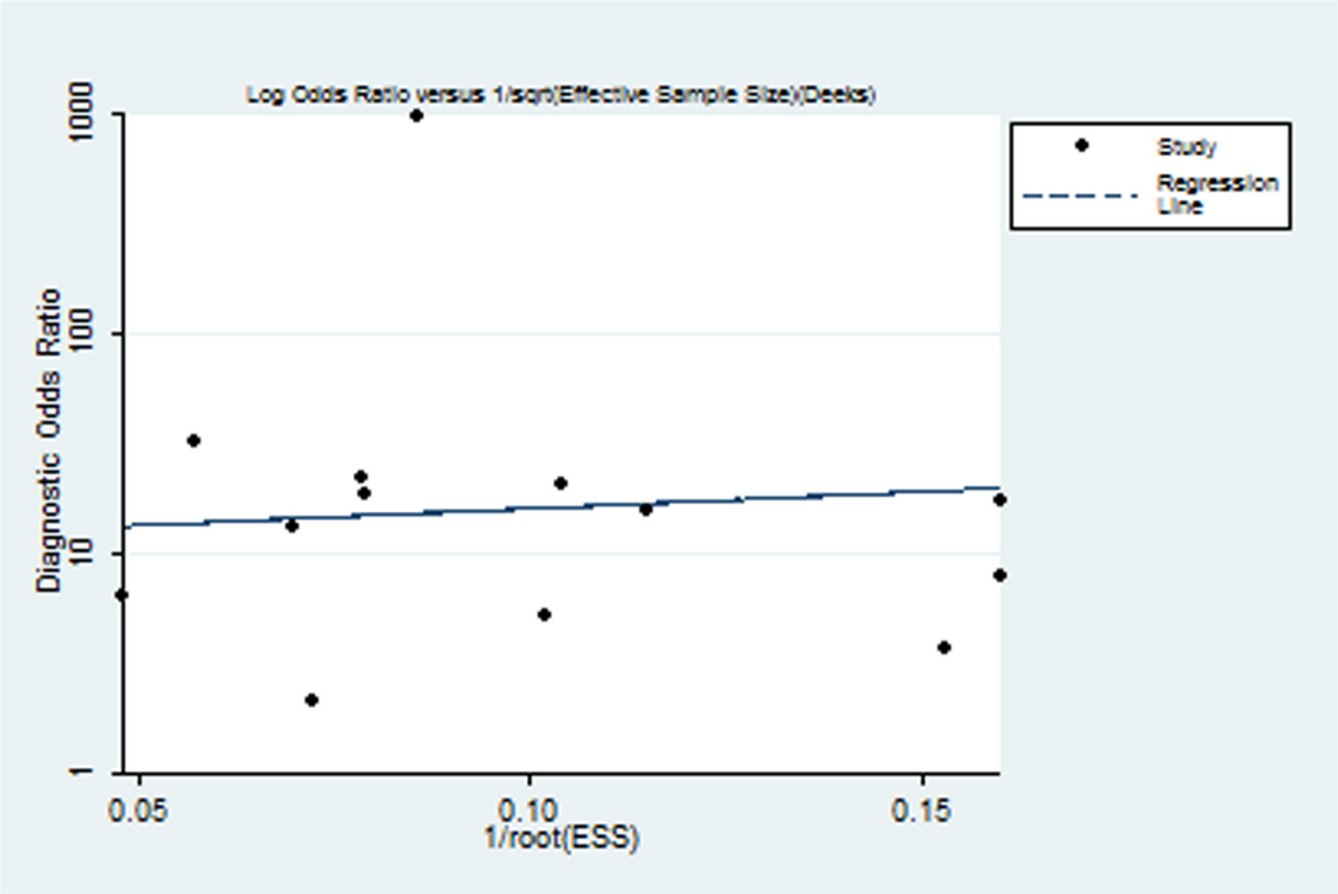
The Funnel plot assessment of potential publication bias. Each solid rectangle represents an eligible study.

The Deek’s test showed that publication bias was not statistically significant (*p* = 0.820). The funnel plots for publication bias were also symmetrical (Fig 5), indicating that publication bias was not significant.

## Discussion

In the current study, we performed a meta-analysis of OPN level in diagnosis of ovarian cancer. The first question we intended to address was whether single OPN is a useful biomarker in diagnosis of ovarian cancer. We found that the overall sensitivity and specificity of OPN in diagnosis of ovarian cancer were 0.66 (95% CI, 0.51–0.78) and 0.88 (95% CI, 0.78–0.93), respectively. The AUC under sROC curve was 0.85 (95%CI, 0.81–0.88). These data indicated that OPN was a useful diagnostic marker for ovarian cancer, although it should be pointed out that one study showed that the OPN level was increased more in the advanced FIGO stages of ovarian cancer [33], suggesting that the diagnostic sensitivity of OPN could be high in advanced ovarian cancer. Kim [22] et al. showed that the diagnostic sensitivity of OPN was 0.80 in early ovarian cancer patients, which is lower than that of advanced patients (0.85). In addition, there was no publication bias observed, indicating that the results are reliable.

Furthermore, our second question intended to address whether OPN could improve the diagnostic accuracy of CA125, a well-established ovarian cancer biomarker, in diagnosis of ovarian cancer. However, we did not compare the diagnostic accuracy of CA125 vs. OPN in diagnosis of ovarian cancer because CA125 is frequently used in the clinic and the test results are not always blinded to gynecologists. Therefore, the test result of CA125 but not OPN may greatly affect the clinical decision of gynecologists. Under such circumstances, the diagnostic accuracy of CA125 may be overestimated [36] and it is unreasonable to compare the diagnostic accuracy of CA125 and OPN. For an undiagnosed ovarian cancer patient, OPN and CA125 can be simultaneously used so that it would be more valuable to determine whether OPN could provide additional information beyond CA125. Statistically, three methods [c-statistics, NRI, and integrated discrimination improvement (IDI)], are currently available to explore whether the index test can add an additional value beyond the traditional test [37]. Among these eligible studies, only one study investigated the added diagnostic value of OPN beyond CA125 by NRI [35]. Although most of the remaining studies investigated the diagnostic accuracy of CA125 for ovarian cancer, they did not statistically confirm whether OPN has an additional diagnostic value beyond CA125. Our data from the current study could conclude that it is still to be elucidated whether OPN improves the diagnostic accuracy of CA125 and a better-designed study is needed to confirm this hypothesis.

The third question we intended to address in the current study was whether OPN is useful in diagnosis of ovarian cancer and we believe our study robustly answers this. We have noted that the major design deficiency of these 13 eligible studies was the subject selection. Ideally, clear inclusion and exclusion criteria should be pre-specified when a test on diagnostic accuracy is performed. These criteria usually consist of medical history and symptoms or signs and are used to outline the characteristics of subjects while representing a group of patients with undiagnostic ovarian cancer. However, we have noted that only one study [26] was prospective design and had clear inclusion and exclusion criteria, while some studies [36, 38, 39] set healthy individuals as the control group. These case-control study designs might over-estimate the diagnostic accuracy of OPN for ovarian cancer. In addition, consecutive or random enrollment is necessary to ensure the prevalence of ovarian cancer in subjects is reflective of that in the real world. However, none of these eligible studies stated that they consecutively enrolled their subjects. Along with subject sampling, it should be noted that some of the eligible studies [24, 27, 28, 30] did not report whether all the subjects received the same reference test, and therefore partial verification bias could not be avoided [40]. Thus, the overall quality of the eligible studies included in this study may not be good enough. We conclude that a future study with a larger sample size, complete verification, clear and unified inclusion and exclusion criteria, and a prospective and consecutive enrollment design is needed to rigorously estimate the diagnostic accuracy of OPN in diagnosis of ovarian cancer.

In addition to pooling the diagnostic characteristics of the index test, identification of heterogeneity is also an important goal of a meta-analysis. Our current study showed that the European origin of patients and controls, and the R&D OPN test Kit were the sources of heterogeneity across these eligible studies. In adition, the publication bias was not significant in the current study, indicating that the results of our meta-analysis are reliable.

The current study does have some limitations. For example, the optimal threshold was obtained from the ROC curve for some studies [27, 31] or scatter plot [28, 30], which may not exactly estimate the actual diagnostic accuracy of OPN. Moreover, twelve of these 13 eligible studies were from Europe or North America, which could yield bias for the specific studied population. Despite these limitations, this is the first systematic review and meta-analysis of the OPN diagnostic accuracy for ovarian cancer. It may guide investigators to better design a future to confirm the potential of biomarkers in diagnosis of ovarian cancer.

## Supporting Information

S1 PRISMA Checklist (DOC)
